# Antibody-Mediated Rejection in Liver Transplantation: Immuno-Pathological Characteristics and Long-Term Follow-Up

**DOI:** 10.3389/ti.2024.13232

**Published:** 2024-08-29

**Authors:** Luca Cicalese, Zachary C. Walton, Xiaotang Du, Rupak Kulkarni, Suimin Qiu, Mohamed El Hag, Heather L. Stevenson

**Affiliations:** ^1^ Division of Transplant Surgery, Department of Surgery, University of Texas Medical Branch, UTMB, Galveston, TX, United States; ^2^ John Sealy School of Medicine, University of Texas Medical Branch, UTMB, Galveston, TX, United States; ^3^ Department of Pathology, University of Texas Medical Branch, UTMB, Galveston, TX, United States; ^4^ Department of Pathology, Cleveland Clinic, Cleveland, OH, United States

**Keywords:** solid organ transplant, allograft, rejection, DSA, AMR, C4d

## Abstract

The diagnosis of liver antibody-mediated rejection (AMR) is challenging and likely under-recognized. The association of AMR with donor-specific antibodies (DSA), and its clinical course in relation to pathologic findings and treatment are ill defined. We identified cases of liver AMR by following the criteria outlined by the 2016 Banff Working Group. Patient demographics, native liver disease, histopathologic findings, treatment type, clinical outcome, and transaminase levels during AMR diagnosis, treatment, and resolution were determined. Patients (n = 8) with AMR average age was 55.2 years (range: 19–68). Seven of eight cases met the Banff criteria for AMR. Personalized treatment regimens consisted of optimization of immunosuppression, intravenous pulse steroids, plasmapheresis, IVIG, rituximab, and bortezomib. Five patients experienced complete resolution of AMR, return of transaminases to baseline, and decreased DSA at long-term follow-up. One patient developed chronic AMR and two patients required re-transplantation. Follow-up after AMR diagnosis ranged from one to 11 years. Because AMR can present at any time, crossmatch, early biopsy, and routine monitoring of DSA levels should be implemented following transaminase elevation to recognize AMR. Furthermore, treatment should be immediately implemented to reverse AMR and prevent graft failure, chronic damage, re-transplantation, and possibly mortality.

## Graphical Abstract

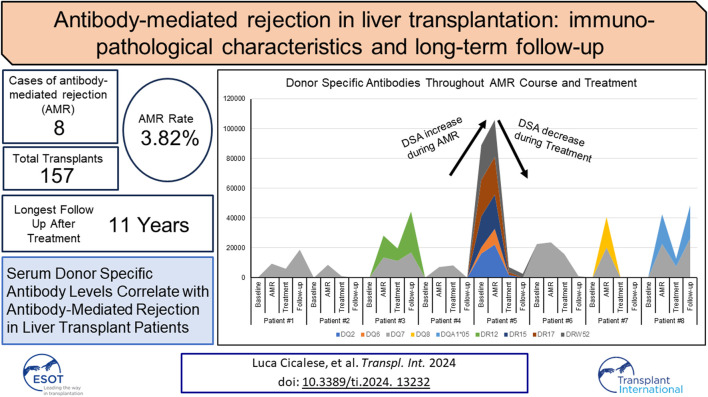

## Introduction

Antibody-mediated rejection (AMR) in liver transplantation (LTX) was first observed in ABO-incompatible recipients caused by preformed or *de novo* donor-specific antibodies (DSA) [1–3]. Over the years, evidence of AMR in ABO-compatible transplants has increased and has sparked increased interest in understanding the pathologic mechanisms and their effect on patient outcomes [4, 5]. Acute AMR occurrence in ABO compatible liver allografts is rare (less than 5%) and is slightly higher in kidney allografts (between 5–10%). Chronic AMR in the liver is less well defined than in kidney allografts, where it is a more common cause of long-term graft loss in approximately 10–20% of transplant recipients [6–9]. The liver is believed to be an “immune-privileged” organ resistant to DSA-mediated injury due to its vast vascular (sinusoidal) endothelial surface, secretion of soluble human leukocyte antigen (HLA) that can bind to and opsonize harmful antibodies and immune complexes, the facilitation of phagocytosis by Kupffer cells, and the presence of a powerful regenerative capacity [10].

The exact incidence of AMR in the liver allograft is likely underestimated. Though increasing evidence and clinical data show that AMR can cause allograft injury and allograft loss, most transplant centers do not monitor DSA prior to LTX and during post-transplant follow-up. Routine HLA testing of donors and recipients as well as lymphocyte crossmatch (CM) is not the standard practice in LTX due to the belief that this organ can absorb and neutralize antibodies with little or no consequence. In recent years, the role of lymphocyte CM, DSA, and complement reactivity, such as C4d, deposition have begun to be recognized as vital markers of graft success or risk of rejection [3, 11–17]. Histopathologic features of AMR in liver allografts, such as portal eosinophilia, portal vein endothelial cell hypertrophy, cholestasis, and microvascular and portal lymphocytic inflammation, while relatively nonspecific in isolation, can be used to recognize acute AMR independent of serology or C4d staining [18]. Additionally, these same histopathological features of AMR are now more recognized when associated with elevated DSA [18, 19].

To establish the Banff schema for histopathological grading of liver allograft rejection, an international consensus group met in 1995 [20], and after several additional meetings, published a comprehensive update and introduced the concept of AMR in 2016 [21]. These guidelines were developed following the presentation and discussion of cases throughout the world at previous Banff meetings over a period of 21 years. These criteria include the presence of serum DSA (>5,000 mean fluorescence intensity), microvascular C4d deposition, compatible histopathologic features (e.g., capillaritis and endothelial cell hypertrophy), and exclusion of other causes that may have similar features [21].

Treatment of AMR in LTX, due to the lack of randomized controlled clinical trials, is mostly adopted from the experience of AMR in kidney transplantation and varies widely among transplant centers [11]. Despite studies recently describing various treatments and outcomes, no gold standard has been established [9, 22]. The mainstay of the management strategies is focused on optimizing immunosuppressors, plasma exchange, IVIG, anti-CD20, and proteasome inhibitors. The current preferred strategy to treat AMR is a personalized (more or less aggressive) approach based on its severity, liver function impairment, DSA levels, and apparent tissue injury on biopsy or the presence of additional risk factors (i.e., infections). Different drugs can be added if the liver disease progresses or if no improvement is seen, or they can be initiated together if the severity of AMR suggests the need to do so. If AMR continues to be present after all medical interventions are exhausted and is associated with worsening liver function and tissue injury, then re-transplantation needs to be considered [23].

In this study cohort, we evaluated CM, DSA levels, applied the 2016 Banff Criteria for the evaluation of liver AMR, and compared the treatment regimens, correlating these findings with long-term clinical outcomes.

## Patients and Methods

### Study Population

The cases were identified and reviewed by interrogating the electronic medical record system (EPIC) and pathology database of 157 patients with LTX performed or followed at the University of Texas Medical Branch (UTMB) from 2009 to 2022. Patients with incompatible ABO donors were not considered as it is well known that ABO incompatibility can significantly increase DSA production and AMR rates [24, 25]. Patients with elevated liver function tests (LFTs), elevated DSA, and biopsy proven AMR according to 2016 Banff Criteria [21] were selected. Histology of AMR cases with elevated DSA were compared to liver biopsies from patients matched by age, sex, same native liver disease, and who did not have elevated DSA. Histology slides were independently reviewed by two transplant pathologists who were blinded to the diagnosis.

Patient demographics, native liver disease, LFTs (prior to the diagnosis, at the time of diagnosis, during treatment, and at most recent follow-up post-AMR episode), and histopathologic findings were noted. The type and duration of treatment and clinical outcome parameters were also analyzed.

Clinical and laboratory data were collected from EPIC according to the Institutional Review Board (IRB) rules and regulations and previous approval of a research protocol (#12-260).

All recipient sera were tested for anti-HLA antibodies using a multiplexed solid-phase-based microbeads array (Single Antigen Class I and II Kits, OneLambda, CA, United States). A pre-transplant serum was considered positive when the mean fluorescence intensity (MFI) was higher than 1,000 (MFI ≥1,000). Additionally, a flow crossmatch (Flow-XM) was conducted using the patient’s serum incubating with donor lymphocytes. The B and T cell flow-XM positivity was defined with a mean channel shift (MCS) >/ = 20 for T cell and >/ = 30 for B cell using a 256-channel resolution on the recipient serum obtained at the time of transplantation.

### Pathology Evaluation

Biopsy sections with hematoxylin and eosin (H&E), special stains including Masson’s trichrome, PAS, PAS-D, iron stain and immunostaining for C4d were re-evaluated for morphologic and immunophenotypic features of acute and chronic AMR according to the 2016 Banff Criteria for allografts (Table 1) [21].

**TABLE 1:** Semi-quantitative histology scores of acute and chronic AMR, adopted from 2016 Banff criteria.

Semi-quantitative histology scores of acute AMR
h-scores (0–3) (0-none, 1-mild, 2-moderate, 3-severe)
Portal microvascular endothelial cell hypertrophy
Portal capillary and inlet venule dilation
Portal microvasculitis
Portal edema
Ductular retention
Cholestasis
Edema and periportal hepatocyte necrosis
Lymphocytic and/or necrotizing arteritis
Moderate portal/periportal, sinusoidal and/or perivenular fibrosis
C4d score (0–3) (0-none, 1-minimal, 2-focal, 3-diffuse)
Mononuclear infiltrates: portal/perivenular/interface (0–3)
(0-none, 1-mild, 2-moderate, 3-severe)
Fibrosis: at least moderate portal/periportal, sinusoidal and/or perivenular (0–3)
(0-none, 1-mild, 2-moderate, 3-severe)
Ductopenia (0–1) (0-none, 1-present)

Two transplant pathologists (H.S.L and S.Q) scored the histology characteristics and C4d staining. The control and study cases were randomly mixed and evaluated by the pathologists who were unaware of the diagnosis. The two pathologists, unaware of other data, assessed biopsies for features including portal microvascular endothelial cell hypertrophy, portal capillary dilatation, dilated or tortuous portal inlet venules, presence of microvasculitis, edema, periportal hepatocyte necrosis and/or lymphocytic arteritis. C4d scores from 0 to 3 were used as recommended in the 2016 Banff criteria. A semi-quantitative grading system was used to demonstrate the histopathological features (Table 1). Final scores were obtained by calculating the average of the scores measured independently by the two pathologists. Graphs were created using Sigma Plot software (SPSS, Chicago, IL) and Excel (Microsoft).

## Results

### Patient Demographics

Patient demographics and native liver diseases including cirrhosis from chronic hepatitis C (HCV), alcohol (ETOH) abuse, alcoholic fatty liver disease, alpha-1 antitrypsin deficiency, and hepatocellular carcinoma (HCC) are summarized in Table 2. Patient race was classified as reported by each patient and listed in EPIC. Among patients receiving a LTX from an identical ABO donor, eight patients were diagnosed with AMR at our institution during the study period. The AMR diagnosis in these patients was established at different intervals from transplantation for each patient. This interval ranged from 12 days to 16 years after transplantation. Two patients had AMR diagnosed within 1 month of transplant, and the others had AMR diagnosed 1, 2, 3, 4, 8, and 16 years after transplant. Two of these patients were followed at our center but received a transplant in another center or state. The rate of AMR observed in the patient population receiving a LTX in our institution was 3.82%. All patients transplanted at our institution received induction with IV basiliximab or methylprednisolone at the time of transplantation and maintenance immunosuppression with tacrolimus and mycophenolic acid and rapid taper to steroid free. The average age of the eight AMR patients was 55.2 years (range: 19–68): four were male and four were female.

**TABLE 2:** Patient demographics.

Patient	Age range	Sex	Race	Cause of native liver disease
#1	61–70	M	White	HCV, ETOH
#2	61–70	F	Black	HCV
#3	21–30	F	White	Alpha-1 Antitrypsin Deficiency
#4	51–60	M	White	HCV
#5	51–60	F	White	HCV, HCC, ETOH
#6	61–70	F	White	HCV, HCC
#7	61–70	M	White	HCV
#8	61–70	M	White	HCV

### Correlation of Lymphocyte Cross Match With Antibody-Mediated Rejection

All patients transplanted at our institution received a CM at the time of LTX. In total, eight positive CM were recorded. However, only two patients with AMR (Patients #5 and #6) had positive CM tests at the time of LTX, and the others were T and B cell negative. Data on the correlation of CM and AMR for individual patients is summarized in Table 3. In patient #5, both B and T cell positivity were detected with a mean channel shift (MCS) >/ = 20 for T cell and >/ = 30 for B cell using a 256-channel resolution on the recipient serum obtained at the time of transplant. DSA alleles A1, A24, B7, B8, DR15, DR17, DR52, DQ2, and DQ6 had mean fluorescence intensity (MFI) values ranging from 2,159–24,404. In patient #6, only B cell positivity was detected on flow cytometry with alleles A1, A24, and DQ7 detected with MFI values of 5,144; 7,586; and 15806, respectively. Patients #5 and #6 with positive CM experienced AMR early during the first-year post LTX. Patients with negative CM experienced AMR several years after transplant (2–4 years after LTX). CM information for patients #3 and #7 were unavailable due to being transplanted elsewhere, and experienced AMR 22 years after receiving a pediatric LTX and 10 years after adult LTX, respectively.

**TABLE 3:** Tacrolimus (TAC) levels with liver injury test profile and serum DSA MFI levels in eight patients who experienced AMR. Values recorded at baseline (post-transplant, but before AMR, or at earliest lab values on file if transplant was not performed at our institution), at the time of AMR diagnosis, during treatment, and at long-term follow up extracted from the most recent lab values on file. Liver injury test profile of patients included monitoring of platelet (PLT) count, total bilirubin (T. Bili), and concentration of the enzymes alanine transaminase (ALT) and aspartate transaminase (AST).

Patient	Time points	Liver injury profile					DSA								
	Follow-up	TAC	PLT	T. Bili	ALT	AST	DQ2	DQ6	DQ7	DQ8	DQA1^a^05	DR12	DR15	DR17	DRW52
	(Years from LTX - years from AMR)	ng/mL	X10^3/µL	mg/dL	U/L	U/L	(SI)	(SI)	(SI)	(SI)	(SI)	(SI)	(SI)	(SI)	(SI)
#1	Baseline	8	233	0.9	30	19			0						
	At AMR Diagnosis	<3	180	11.6	138	63			9,209						
	Treatment	6	164	0.9	76	47			5,980						
	Follow-Up (13-9)^c^	3	239	0.5	25	29			18,807						
#2	Baseline	<3	193	1	29	26			0						
	At AMR Diagnosis	13	150	5.3	101	163			8,665						
	Treatment	9	102	4.4	50	96			863						
	Follow-Up (13-11)	na	144	0.5	13	25			0						
#3	Baseline	4	185	0.8	56	48			NA			NA			
	At AMR Diagnosis	7	101	1.4	87	119			13,291			14,873			
	Treatment	8	110	1	54	69			11,308			8,200			
	Follow-Up (17-1)^b^	8	90	0.6	49	57			16,820			27,512			
#4	Baseline	8	211	0.8	40	36			0						
	At AMR Diagnosis	11	184	1.2	59	52			7,074						
	Treatment^d^	11	300	0.4	28	33			8,333						
	Follow-Up (8-5)	<3	286	0.6	23	31			NA						
#5	Baseline^a^	12	108	5.1	74	26	16,092	4,114					20,884	24,404	23,464
	At AMR Diagnosis	7	175	6.6	56	23	22,197	10,408					23,066	25,182	24,941
	Treatment	7	120	0.9	33	32	1,619	0					0	1901	3,476
	Follow-Up (6-6)	3	105	0.8	25	37	0	0					0	0	2,592
#6	Baseline^a^	6	292	0.7	88	36			22,348						
	At AMR Diagnosis	5	188	1	325	162			23,469						
	Treatment	6	84	1.9	38	29			1,221						
	Follow-Up (4-4)	8	175	0.6	11	21			1,088						
#7	Baseline	7	208	1.1	119	62			NA	NA					
	At AMR Diagnosis	7	172	4.9	434	203			20,172	20,495					
	Treatment	7	242	1.1	211	91			NA	NA					
	Follow-Up (11-3)^b^	6	295	2.1	83	51			NA	NA					
#8	Baseline	10	876	0.5	42	47			0		0				
	At AMR Diagnosis	7	643	12	921	622			22,514		20,089				
	Treatment	8	551	8.6	170	132			7,575		5,119				
	Follow-Up (3-2)	6	598	0.5	23	24			26,110		22,373				

^a^ Represents a positive crossmatch at time of transplant.

^b^ Represents patients requiring re-transplantation.

^c^ Represents patients experiencing chronic AMR.

^d^ Represents patients with history of noncompliance with immunosuppressive medications.

NA represents lab values that were unavailable.

### DSA Correlation With AMR

**FIGURE 1:**
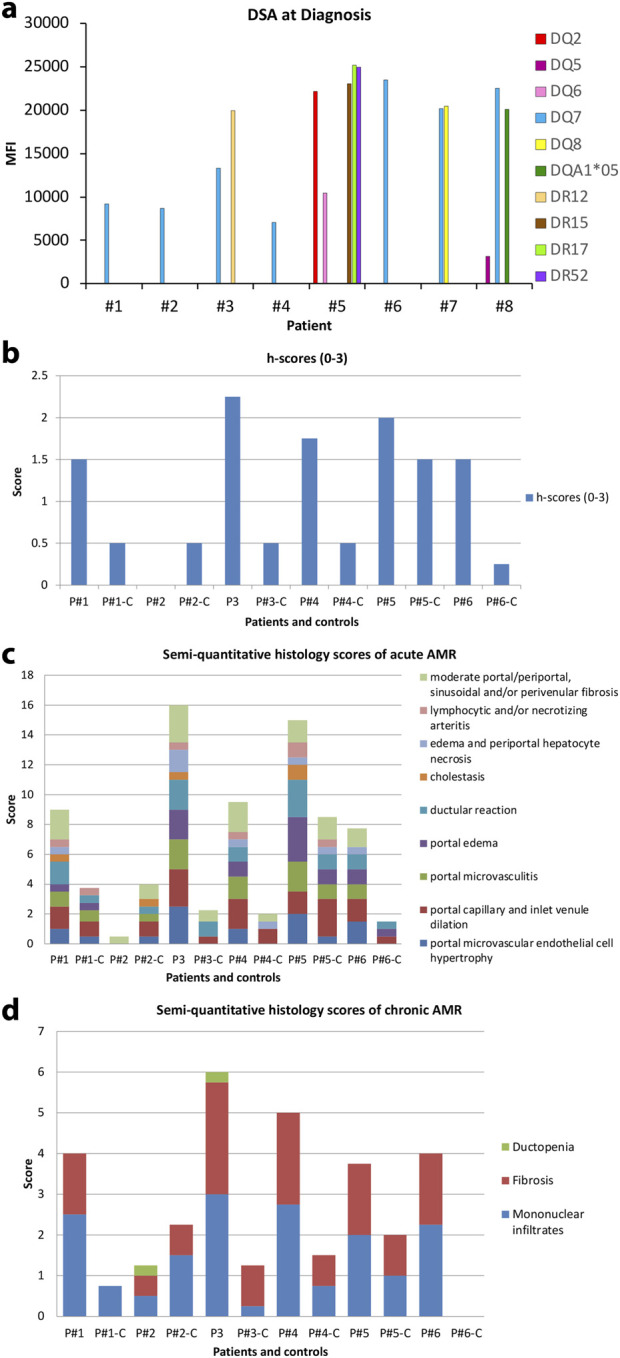
**(A)** Serum DSA levels at the time of diagnosis of AMR in the eight patients. **(B)** h-score of eight AMR patients according to 2016 Banff Criteria for liver allografts. Of these, seven out of eight patients are greater than one. Patient #2 showed minimal pathologic changes in liver biopsy and h-score is zero. **(C)** Semi-quantitative histology scores of acute AMR in patients where “C” denotes matched control patients. **(D)** Semi-quantitative histology scores of chronic AMR in patients “C” denotes matched control patients.

The presence of preformed or *de novo* HLA DSA has been previously associated with rejection, inflammation, fibrosis, and allograft loss in liver transplants [13, 26, 27]. DSA are one of the four criteria to diagnose AMR. In patients with a negative CM experiencing AMR, class I DSA were detected in only one patient while all had high levels of one or more class II DSA. In this patient, class I DSA allele CW4 had MFI of 1,234 several days after diagnosis of AMR, but shortly returned to zero and was not recorded again. HLA class II DSAs with MFI >5,000 at the time of AMR diagnosis alongside the values at baseline, after AMR treatment, and at the most recent follow-up are listed in Table 3. Two out of eight patients had baseline Class II DSA level positivity and B cell positive CM as described above. DSA baseline levels were unavailable in the two patients transplanted elsewhere. Class II DSA, most commonly against the DQ and DR loci, were elevated to MFI >25,000 at the time of diagnosis and decreased after treatment in all cases for which data was available (Figure 1A). Among the DSA elevated at diagnosis (Figure 1A), DSA against DQ7 was present in 5 out of 8 patients. The single patient without DQ7 antibodies showed multiple Class II DSAs against other loci with high MFI levels.

### Histological Correlation With AMR

Two transplant pathologists reviewed the randomized liver biopsies without knowledge of any clinical or serological data from patients who experienced liver allograft AMR and control patients. Histopathologic features were graded with an h-score according to the 2016 Banff Criteria (Figure 1B). Seven out of eight patients received an h-score greater than 1, while all eight control patients received an h-score no more than 1. Patient #2 showed minimal pathologic changes in liver biopsy and received an h-score of zero. All biopsies from matched control patients showed minimal histopathologic changes except control patient #5 which showed relatively active and similar pathologic changes with AMR patients. A semi-grading system as a supplemental tool to h-score system adopted from 2016 Banff Criteria was utilized to demonstrate the break-down of histopathologic features of acute and chronic AMR (Figures 1C, D). Portal microvascular endothelial cell hypertrophy, portal capillary and inlet venule dilation, microvasculitis, portal edema, ductular reaction, cholestasis, periportal hepatocyte necrosis, lymphocytic and/or necrotizing arteritis, portal/periportal, sinusoidal and/or perivenular fibrosis have been carefully evaluated on each biopsy. Ductopenia, fibrosis, and portal and perivenular mononuclear infiltrates were evaluated for active chronic AMR. Figure 1C summarizes the classic histopathologic features observed in the eight AMR patients compared with the mild and unspecific histopathologic changes seen in the control patients. Figure 1D shows the histopathologic features evaluated for chronic AMR. Though the h-scores of the AMR patient group were higher than the control group, the difference between the two group was minimal. The biopsy of patient #2 shows minimal histologic changes compared to the matched control patient.

**FIGURE 2:**
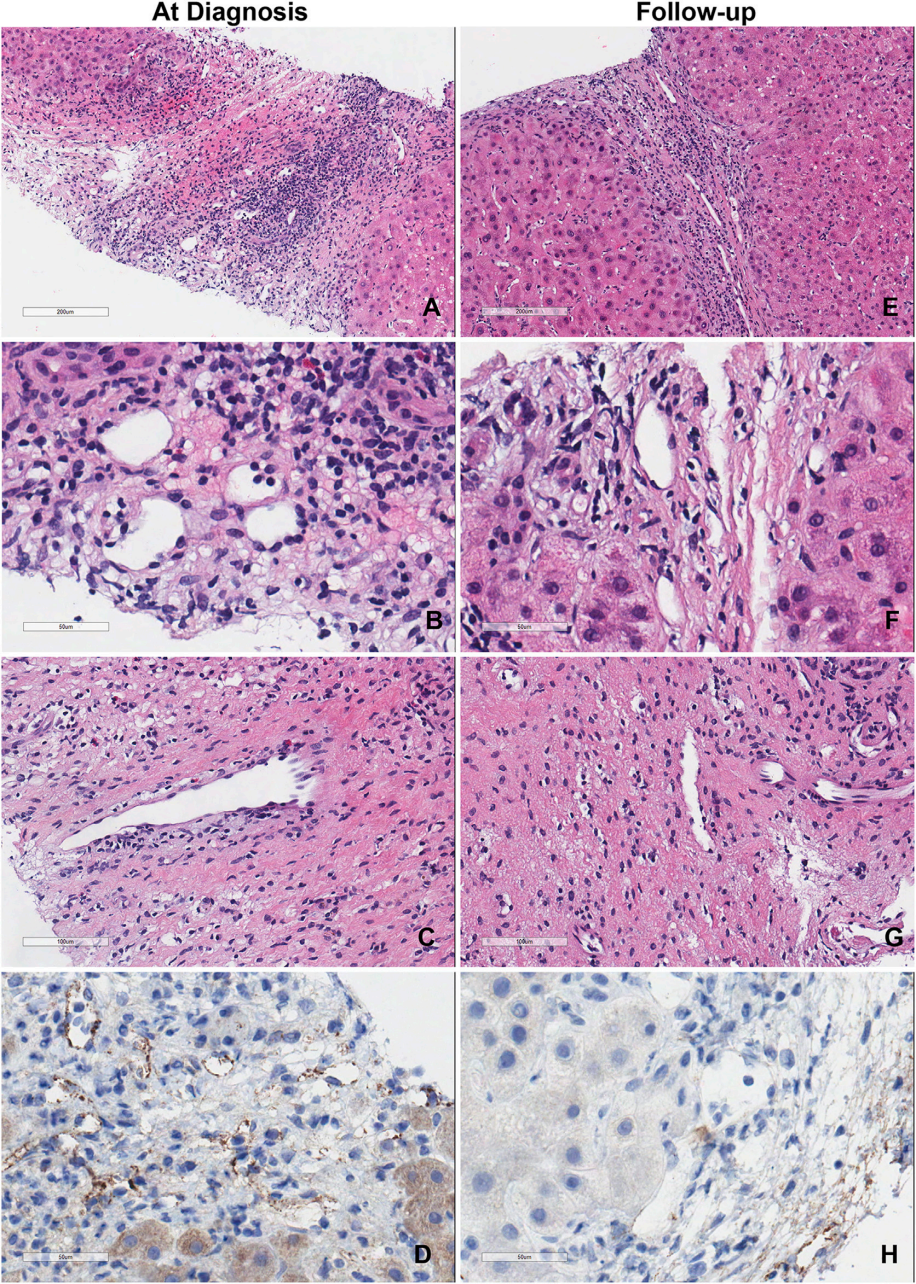
Representative liver biopsy histology of patient #3 at diagnosis and 6-month follow-up. **(A–D)** Liver biopsy of the patient at the time of diagnosis showed the classic acute AMR microvascular pathology lesions. **(E–H)** Liver biopsy of the same patient at time of 6-month follow-up.

Figure 2 shows the representative liver biopsy histology of patient #3 at the time of diagnosis and 6-month follow up. Endothelial cell hypertrophy, capillary and inlet venule dilatation, mixed portal inflammation, portal edema and ductular reaction were observed. Though serum DSA was persistent in patient #3, the histopathology of the liver biopsy at 6-month follow-up improved substantially.

**FIGURE 3:**
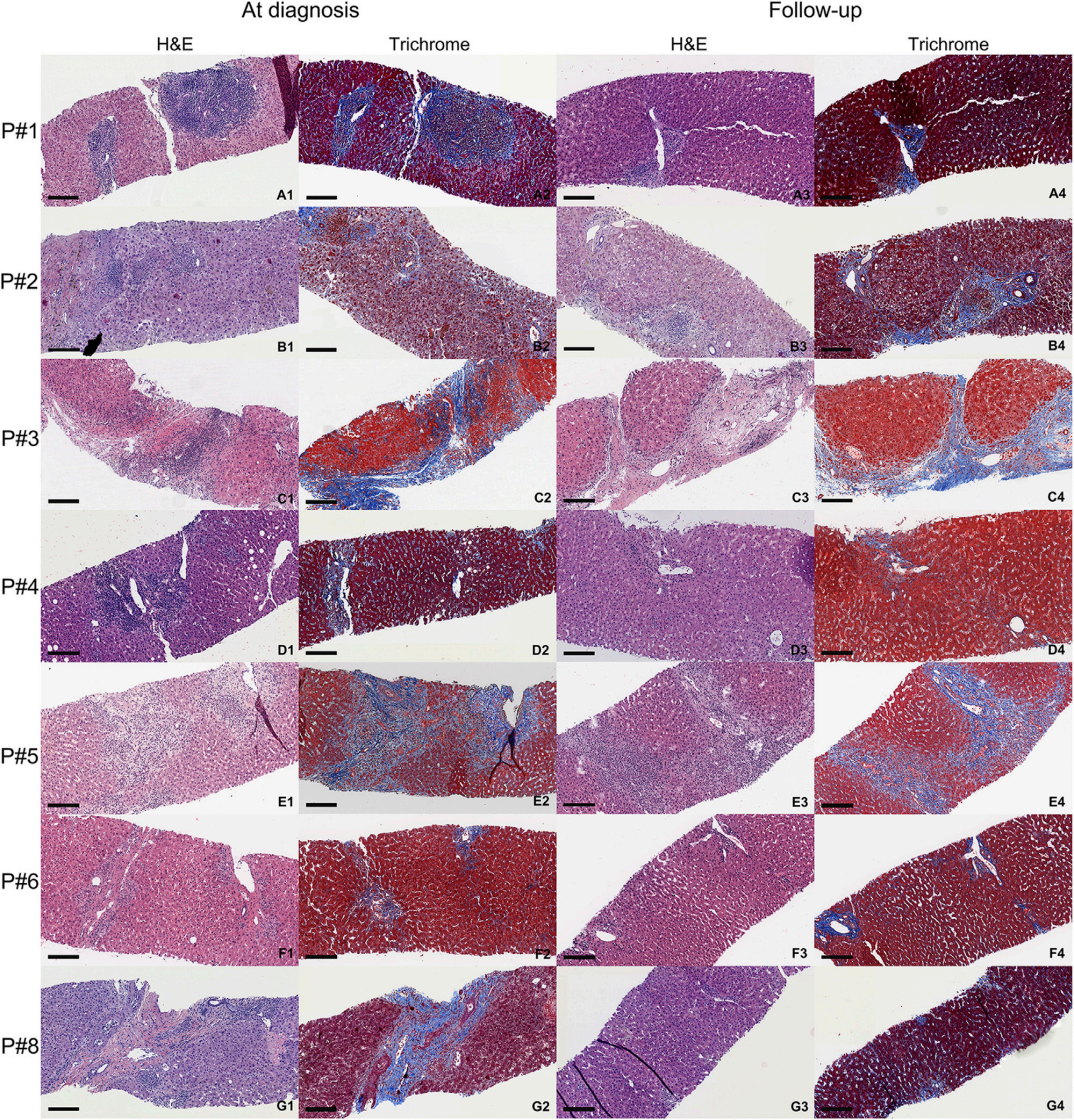
Representative histology of AMR patients at the time of diagnosis (first two columns) and 6–12 months follow-up (last two columns). Each biopsy shows H&E and Masson’s trichrome stain (fibrotic areas are blue). Black scale is 200 µm. **(A1–A4)** are the biopsies from AMR patient #1. **(B1–B4)** are the biopsies from AMR patient #2. **(C1–C4)** are the biopsies from AMR patient #3. **(D1–D4)** are the biopsies from AMR patient #4. **(E1–E4)** are the biopsies from AMR patient #5. **(F1–F4)** are the biopsies from AMR patient #6. **(G1–G4)** are the biopsies from AMR patient #8. Patient #7 did not have a follow up biopsy after diagnosis.

Representative histology in biopsies of AMR patients at the time of diagnosis and 6–12 months follow up are summarized in Figure 3. Masson’s trichrome stains of the biopsies at the time of diagnosis and follow up are also included in Figure 3 to compare the amount of fibrosis. Follow-up liver biopsies of AMR patients showed improvement and returned to baseline after treatments in 6–12 months.

### C4d Scores Correlated With AMR

Positive microvascular endothelial cell C4d staining is one of the key diagnostic criteria for AMR in the liver allograft. C4d deposition in the liver biopsy of the AMR patients was scored using the Banff Criteria. C4d staining in portal veins, portal capillaries, portal stroma, sinusoidal and central vein endothelium was graded as negative (score 0), minimal (<10%, score 1), focal (10%–50%, score 2) and diffuse (>50%, score 3). Seven out of eight AMR patients had a C4d score greater than one. Patient #2 with minimal histologic changes had negative C4d staining.

### Bilirubin and Transaminase Levels and Correlation With AMR

**FIGURE 4:**
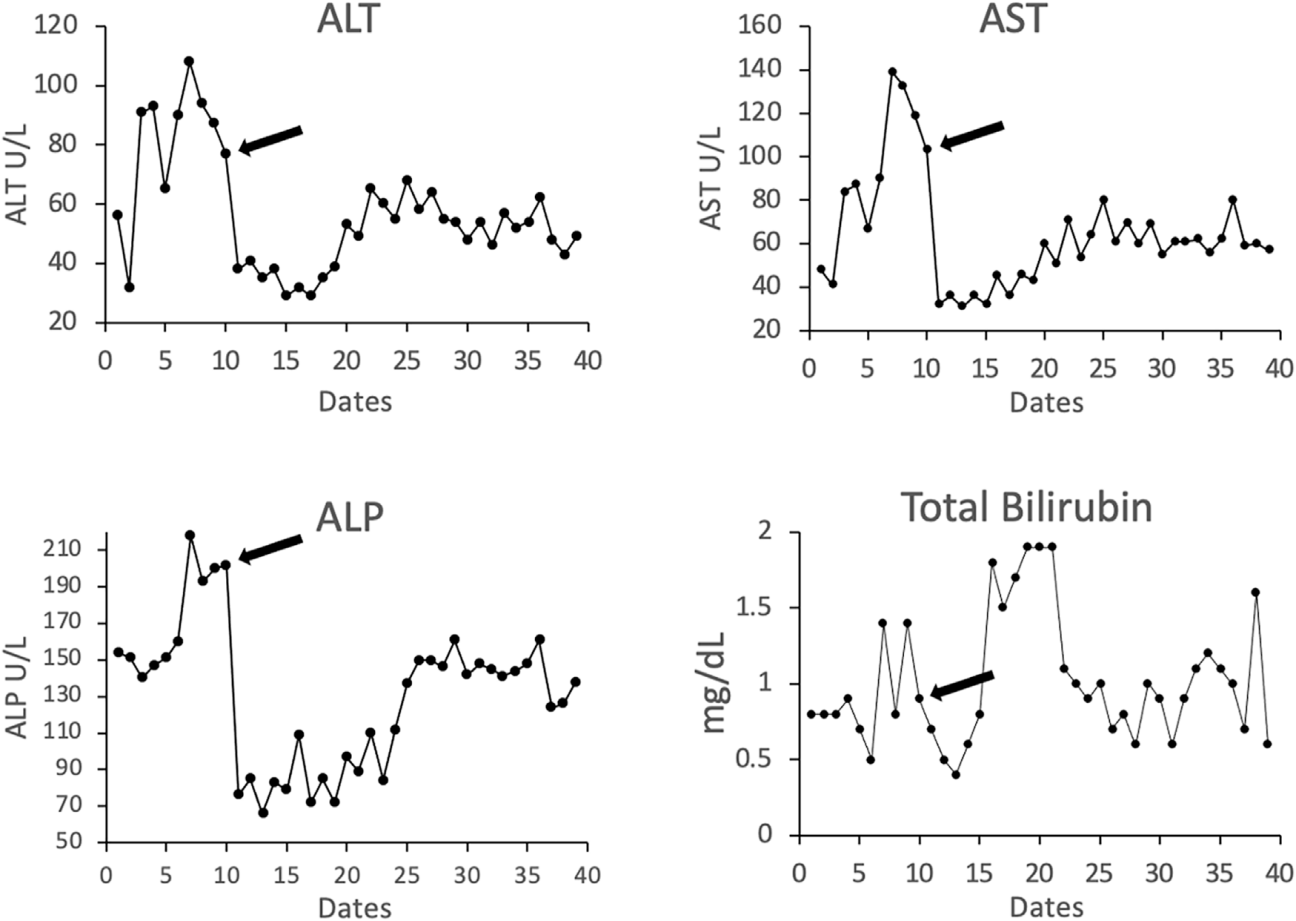
Representative liver-related laboratory testing of total bilirubin and the enzymes alanine transaminase (ALT), aspartate aminotransferase (AST), and alkaline phosphatase (ALP) in patient #3. Arrow indicates the time point at the start of treatment.

Laboratory tests including total bilirubin (T. Bili), alanine transaminase (ALT), aspartate transaminase (AST), and platelets (PLT) have been summarized at baseline 1-2 months after LTX, when AMR was diagnosed, and at last available follow-up. The number of years from transplant and from the AMR episode are also summarized in Table 3. Patient’s liver function returned to baseline after treatments. Figure 4 shows the representative trending of liver function of patient #3.

### Treatment Regimens for AMR in Liver Transplant Recipients

The treatment regimens used in these eight liver transplant recipients with AMR are listed in Table 4. According to our experience, an individualized combination of the treatments was implemented. Treatment regimens varied from using only IV steroid with simultaneous increase of tacrolimus and mycophenolic acid dose to a more aggressive combination of steroids, IVIG, plasma exchange, bortezomib and rituximab.

**TABLE 4:** Immunosuppression treatments utilized for antibody-mediated rejection (AMR).

Patient	IVIG	TPE	Steroids	Bortezomib	Rituximab
1	Yes	Yes	No	Yes	No
2	No	Yes	No	Yes	Yes
3	Yes	Yes	Yes	Yes	Yes
4	Yes	No	No	No	No
5	Yes	Yes	No	Yes	Yes
6	No	No	Yes	No	No
7	No	No	Yes	No	No
8	Yes	Yes	Yes	No	No

### Outcomes and Long-Term Follow-Up

Five patients with liver AMR had complete resolution, return of transaminases to baseline and decreasing DSA levels at follow-up. One patient (Patient #1) developed chronic AMR and two patients (Patients #3 and #7) required re-transplantation. Of the patients requiring re-transplantation, one did not have concomitant T cell-mediated rejection (TCMR), and the other only had a mild TCMR component that was not responsible for the graft loss. Re-transplantation was indicated due to AMR. After the AMR episode and treatment, these patients were followed long-term with periodic DSA monitoring. Up to the latest follow-up of this study in April 2022 (range of follow-up from LTX 4–23 years and from AMR episode 1–9 years), no additional acute AMR episodes were recorded and all patients’ liver laboratory tests continued to remain within normal range and stable. DSA levels remained stable after normalization except for one case (patient #1) who had elevated DQ7 9 years after the initial episode of AMR (PI = 18,807) but with no evidence of AMR and with normal liver function. Of these patients, only one patient (Patient #4) is now deceased.

## Discussion

Analysis of LTX patients at our institution revealed that 3.82% of LTX patients experienced AMR, which is on the higher end of the expected 2%–5% range [9]. It is possible that this higher rate of AMR observed could be secondary to more aggressive monitoring instituted in our program. In fact, in addition to the CM we also frequently measure DSA levels when there is an increase of LFTs, or suspected rejection, either TCMR or AMR. In numerous occasions, TCMR (mild or moderate) was diagnosed without DSA variation and no histological evidence of AMR. However, patients with elevated DSA had AMR as histologically confirmed using the Banff Criteria as previously described. In one patient, AMR occurred 6 months after a successfully treated episode of TCMR and another patient had concomitant histological findings of TCMR and AMR.

In our center, the induction immunosuppressive treatment has been performed with basiliximab, 40 mg at time of transplant and a second dose of 20 mg on post-operative day (POD) 4 and/or methylprednisolone 500 mg at the time of transplant followed by a taper to steroid free in the following week. Thymoglobulin or alentuzumab induction were not used in our program. Maintenance immunosuppressive therapy was performed with dual therapy of oral tacrolimus and mycophenolic acid. This possibly less aggressive induction therapy was utilized to limit post-operative infectious complications and better control hepatitis C virus (HCV) infection prior to the availability of the new and more effective antiviral treatments and reduce the risk associated with COVID-19 in recent years.

The higher rate of AMR observed could be also secondary to this immunosuppressive approach. However, we have no direct evidence of this since most patients experienced AMR several years after transplant and, therefore, when the effects from stronger induction would have faded away [29–31]. Two of the patients who had AMR in the first-year post-transplant also had a positive CM as previously described. Therefore, this observation should trigger the selection of a different induction or maintenance therapy with a higher drug dose/level or steroids to possibly mitigate such risk. The CM results and induction therapy used were unknown for the two patients originally transplanted elsewhere; however, they were maintained on similar maintenance therapy as the other patients in this study. Because of this, it is difficult to draw conclusions, and a clinical trial with a larger number of patients is warranted to establish strong therapeutic indications. However, the correlation of early AMR and positive CM indicates these patients should receive more aggressive graft monitoring with DSA measurements and early biopsy including C4d staining when LFTs rise. Additionally, six out of eight patients with positive CM did not develop AMR during the post operative follow up.

In this study, and similarly to what is observed in kidney allografts, a negative CM at the time of transplant does not appear to exclude the possibility to develop AMR later as we observed in several patients and as was previously described [32]. Therefore, from our limited observations, we conclude that positive CM is not predictive of AMR, but when AMR develops in patients with positive CM it appeared earlier and was more severe.

As stated above, TCMR was associated with, or temporarily preceded, AMR. This can be explained with a secondary stimulation of plasma cells and antibody production from an initial T cell response. Such clinical observations in LTX patients indicates regular monitoring of DSA and C4d measurements similar to what is performed in kidney transplants to rule out AMR in liver allografts [11].

Treatment of AMR varied in our experience. It was individualized based on the severity of the clinical findings ranging from IV steroids to an aggressive combination of plasmapheresis, and Rituximab. The two patients requiring re-transplantation were treated, one with IV methylprednisolone and the other with a combination of IV steroids, TPE, IVIG, bortezomib, and rituximab. The one patient who developed chronic AMR received IV methylprednisolone, IVIG, TPE, and Bortezomib. These results indicate that despite complete resolution of AMR and DSA in five out of eight patients (62.5%) the remaining were probably either undertreated, suggesting that a more aggressive therapeutic approach should be implemented early upon diagnosis of AMR, or resistant to treatment. For patients #3 and #7 it is difficult to evaluate since both received LTX and received immunosuppressive management outside our facility prior to AMR diagnosis. Having received their LTX outside of our facility, it is unknown how long they were experiencing AMR before they presented to us, which supports the idea that more aggressive immunosuppressive treatment should be implemented early in the diagnosis of AMR, and further suggests that LTX patients should be regularly evaluated and tested for DSA and evaluated for liver biopsy evidence of AMR if LFTs rise. Importantly, we observed that earlier intervention with increased immunosuppression following AMR diagnosis resulted in quicker resolution of AMR episodes. Thus, the exact immunosuppressive regimen does not appear to be as important for AMR resolution as the timing of the intervention. The importance of early diagnosis and treatment implementation is supported by other authors [23, 33].

We observed that mainly HLA Class II DSA were identified in these patients. Of the HLA Class II DSA, DQ (especially allele DQ7) antibodies were more clinically relevant to diagnose AMR. No Class I DSA were detected in any of these patients during these rejection episodes. In at least 2 patients, there were *de novo* antibodies, and in four patients there were preformed antibodies. In two patients (Patients #3 and #7), the lack of baseline data does not allow us to determine *de novo* or preformed DSA levels prior to AMR diagnosis at our institution. The two patients with positive CM had preformed DSAs: high levels of DQ7 with positive B cell CM in one case and several DRs but no DQ resulting in T and B cell positivity in the other case. However, our findings are in line with other studies indicating that Class II DSAs play a role in determining graft survival and AMR [34–37].

In long-term follow-up, most patients responded to treatment with complete resolution of AMR as evidenced by the return of LFTs to baseline and lack of histological evidence of AMR. However, in some cases the DQ family of class II DSA remained persistently elevated similar to other studies [33, 38]. The significance of this finding could be explained by a possible neutralization of the present DSA by the “primed and regenerating” liver parenchyma after AMR without consequent evident clinical injury, basically a form of chronic subclinical AMR, but this remains largely unexplained [39–41].

Limitations of this study include small sample size from a single center and non-standardized treatment regimens. Further studies involving a larger number of patients from multiple centers are needed to corroborate our findings. Additionally, whether or not certain immunosuppressive medications may be more adept for treatment of AMR episodes is currently unknown. As mentioned previously, it appears that swift implementation of immunosuppression following AMR diagnosis is sufficient regardless of treatment regimen. However, additional studies involving different treatment regimens for AMR are indicated to determine optimal medications and treatment time, length, and intensity for AMR resolution and overall graft survival.

From the observations made in our series of patients, we can conclude with confidence that AMR is a clinically underestimated and underdiagnosed entity in LTX recipients. The current Banff Criteria, albeit conservative, is well accepted and is an important diagnostic tool in the identification of AMR in LTX patients. AMR can present at any time, including many years after transplant or possibly earlier if a positive CM was detected at the time of transplant, or if the patient is non-compliant with taking immunosuppressive medications. More aggressive monitoring with DSA measurement, especially DQ and DR, as well as early biopsy and C4d staining should be routinely implemented when LFT elevation is observed and when TCMR is suspected/identified to recognize AMR [19]. Consequently, treatment should be immediately implemented to completely reverse AMR and to prevent graft failure, chronic damage, re-transplantation, and possibly mortality in this patient population.

## Data Availability

The original contributions presented in the study are included in the article/Supplementary Materials, further inquiries can be directed to the corresponding author.

## Abbreviations

ALP, Alkaline Phosphatase; ALT, Alanine Transaminase; AMR, Antibody-Mediated Rejection; AST, Aspartate Transaminase; CM, Crossmatch; DSA, Donor Specific Antibody; FFPE, Formalin-Fixed and Paraffin-Embedded; H&E, Hematoxylin & Eosin; HCV, Hepatitis C Virus; HLA, Human Leukocyte Antigen; IRB, Institutional Review Board; LFT, Liver Function Test; LTX, Liver Transplantation; MCS, Mean Channel Shift; MFI, Mean Fluorescence Intensity; PLT, Platelet; T. Bili, Total Bilirubin; TAC, Tacrolimus; TCMR, T Cell-Mediated Rejection.
